# Comparison of Random Survival Forest Based‐Overall Survival With Deep Learning and Cox Proportional Hazard Models in HER‐2‐Positive HR‐Negative Breast Cancer

**DOI:** 10.1002/cnr2.70262

**Published:** 2025-07-07

**Authors:** Wenqi Cai, Yan Qi, Linhui Zheng, Huachao Wu, Chunqian Yang, Runze Zhang, Chaoyan Wu, Haijun Yu

**Affiliations:** ^1^ Department of Radiation and Medical Oncology, Hubei Key Laboratory of Tumor Biological Behaviors, Hubei Cancer Clinical Study Center Zhongnan Hospital of Wuhan University Wuhan Hubei China; ^2^ Xinzhou Traditional Chinese Medicine Hospital Zhongnan Hospital of Wuhan University (Xinzhou) Wuhan Hubei China; ^3^ Department of Integrated Traditional Chinese Medicine and Western Medicine Zhongnan Hospital of Wuhan University Wuhan Hubei China

**Keywords:** breast cancer, CoxPH, Deepsurv, RSF, RSF‐VIMP

## Abstract

**Background:**

Traditional CoxPH models are limited in handling real‐world data complexities. While machine learning models like RSF and DeepSurv show promise, their application and comparative evaluation in the HER2‐positive/HR‐negative breast cancer subtype require further validation.

**Aims:**

This study aims to build a survival prediction model for breast cancer patients based on different methods. The optimal model will provide more accurate survival predictions for clinical decision‐making of HER2 positive and HR negative cancer patients.

**Methods and Results:**

This study analyzed 8,119 HER2‐positive HR‐negative breast cancer patients from the SEER database, randomly allocated to training/validation/test cohorts (7:1:2 ratio). Predictive models were developed using five feature sets and three algorithms (Cox PH, RSF, DeepSurv), with feature selection optimized via Concordance index (C‐index). Evaluation revealed: The C‐index of the DeepSurv models constructed using the training set is greater than 0.8, performing better than both the RSF and CoxPH models. However, CoxPH outperforms DeepSurv in terms of C‐index when testset. The Brier scores for all models were below 0.25. Which indicates that the models predicted with high accuracy. Based on the training set, the Deepsurv model predicted the highest ROC‐AUCs of 0.91, 0.863, and 0.855 for 1‐, 3‐, and 5‐year overall survival (OS), respectively. The RSF model achieved the highest AUCs, specifically 0.876, 0.861, and 0.845, for 1‐, 3‐, and 5‐year overall survival in the test group. The calibration graphs indicate that of the three models forecasting overall survival at 1, 3, and 5 years, the RSF model demonstrated the greatest level of agreement between predictions and actual observations, trailed by the DeepSurv model. There was poor agreement between CoxPH model predictions and observed data. Optimal Clinical Net Benefits at 1, 3, and 5 Years for DCA of the Deepsurv Model in the Training Set Data. However, in the test set, compared to other models, RSF showed better Optimal Clinical Net Benefits.

**Conclusions:**

In conclusion, compared to conventional prognostic models, the Random Survival Forest (RSF) model serves as a reliable tool for predicting long‐term survival in breast cancer patients, demonstrating consistent performance across diverse datasets. Furthermore, the feature set selected via RSF‐Variable Importance (VIMP) (compared with LASSO regression and Cox regression) significantly enhances the performance of prognostic models. Our findings may offer practical guidance for future development of long‐term prognostic models tailored to breast cancer subtypes.

## 1 Introduction

Breast cancer is a heterogeneous disease with a wide variety of genotypic and phenotypic characteristics [1]. Human epidermal growth factor receptor 2 (HER2) overexpression in primary breast cancer is associated with a poor prognosis in 15%–20% of cases, and almost 50% of HER2‐positive breast cancers do not express hormone receptors (HRs) [2]. Survival rates are greater in cases of HR‐positive/HER2‐positive breast cancer compared to those with HR‐negative/HER2‐positive breast cancer [3]. HER2‐positive breast cancer comprises four molecular subtypes (single HR‐positive and double HR‐positive/double HR‐negative) with distinct clinical and biological characteristics [4]. Although HER2‐positive/HR‐positive breast cancer has been extensively studied, the demographic characteristics, clinical pathological features, and survival outcomes of patients with HER2‐positive/HR‐negative breast cancer remain insufficiently elucidated [5]. This poses challenges for individualized clinical management. Therefore, constructing a prognostic model for the HER2‐positive/HR‐negative breast cancer subgroup is of significant importance.

One of the most commonly used approaches in survival analysis is the traditional proportional hazards model (CoxPH). The CoxPH model is a linear regression model. Fan et al. [6] developed a patient survival model using multivariate CoxPH of 34 819 cases of HER2‐positive breast cancer in the SEER database (C‐index = 0.853). Liu et al. [7] similarly modeled the survival of 7203 young breast cancer patients (C index = 0.783). However, CoxPH models may be too simple to fit real‐world data because real‐world data typically do not satisfy the linear proportional risk condition. Random Survival Forest (RSF), a machine learning‐based predictive model, is a decision tree integration method that estimates cumulative hazard functions. It has been demonstrated to outperform traditional predictive models in terms of predictive performance [8, 9]. Li et al. [10] have demonstrated that the RSF model outperformed conventional CoxPH models in predicting long‐term survival of breast cancer patients. In addition, Katzman et al. [11] proposed the DeepSurv model, which integrates the CoxPH framework with a deep learning neural network. When dealing with complex treatment‐related prognostic analyses, the DeepSurv model significantly outperforms traditional regression methods.

In this study, we employed three methodologies—the Cox proportional hazards (CoxPH) model, Random Survival Forest (RSF), and a neural network model (DeepSurv)—to predict long‐term survival outcomes in specific breast cancer subpopulations using data from the SEER database. Model performance was systematically compared through C‐index in conjunction with three feature selection strategies: LASSO regression, Cox regression, and RSF‐Variable Importance Measure (RSF‐VIMP). Additionally, we identified the most effective feature selection scheme across the models and further validated the model performance through Brier scores, Receiver Operating Characteristic—Area Under the Curve (ROC‐AUC) analysis, and decision curve analysis (DCA). These results provide actionable insights for refining prognostic models tailored to breast cancer subtypes, emphasizing the integration of robust feature selection and model interpretability in future research.

## 2 Materials and Methods

### 2.1 Data Source

We used SEER*Stat 8.4.0.1 (National Institutes of Health, Bethesda, MD) to obtain individual breast cancer data published in the SEER database from 2010 to 2015. These data included demographic, clinicopathologic, and survival information. Our study included 81 783 breast cancer patients who were initially identified from the SEER database. The SEER database started collecting HER2 status in 2010. Therefore, the year of breast cancer diagnosis was considered to be 2010. After removing missing data and PR‐ and ER‐positive patients, a total of 8119 HER2‐positive HR‐negative breast cancer patients were included in this cohort study. Demographic characteristics were extracted: age at diagnosis, sex, and race. Clinical characteristics included bone metastasis status, lung metastasis status, liver metastasis status, brain metastasis status, tumor stage, tumor grade, histologic type, T stage, N stage, M stage, and months of survival.

Patients were categorized into age groups of less than 30 years, 30–39 years, 40–49 years, 50–59 years, 60–69 years, 70–79 years, and 80 years and older. Race was categorized as white, Asian or Pacific Islander, and American Indian/Alaska Native. Tumor grades were classified as well‐differentiated (Grade I), moderately differentiated (Grade II), poorly differentiated (Grade III), and undifferentiated (Grade IV). Patients were grouped according to T‐stage (T0, T1, T2, T3, T4), N‐stage (N0, N1, N2, N3), and M‐stage (M0, M1). The histologic type was described as invasive ductal carcinoma (IDC) [International Classification of Diseases of Oncology, Third Edition (ICD‐O‐3) code 8500/3], invasive lobular carcinoma (ILC) (ICD‐O‐3 code 8520/3), mixed IDC and ILC (ICD‐O‐3 code 8522/3), or other types, according to the ICD‐O‐3 pathology codes.

The informed consent from patients was waived because of the public nature of the SEER database by the use agreement (ID: 18864‐Nov2021). Ethics approval was not required for this study.

### 2.2 Statistical Analysis

**FIGURE 1:**
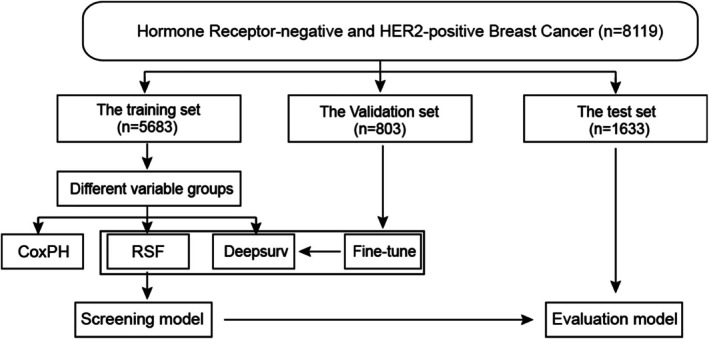
Flowchart for data analysis.

The study analyzed the clinical characteristics of patients diagnosed with HER2‐positive HR‐negative breast cancer (Table 1). Overall survival (OS) was defined as the interval from diagnosis to breast cancer‐related or all‐cause mortality. Kaplan–Meier survival curves were used to assess OS. Log tests were used to assess differences in survival. Univariate Cox proportional risk regression analyses were conducted to determine the hazard ratios (HRs) and 95% confidence intervals (CIs) for OS. Multivariate Cox regression models were used to calculate HRs and 95% CIs for OS. Evaluate models using multivariate Cox regression and select the set of features linked to significantly different Cox models (Cox 10). The LASSO regression produced the feature set (LASSO 8), while the Random Survival Forest model was built using the Random Forest SRC R package. Based on variable importance, two feature sets (RSF 12 and RSF 14) were acquired. We performed deep learning computations to predict survival outcomes for breast cancer patients based on the DeepSurv computational method described by Katzman et al. [11]. The data from the training cohort was utilized to construct the DeepSurv model for the seven‐layer neural network, while the validation set was employed to refine the model parameters. Then, we assess models built with diverse feature sets using the test set data and calculate their C‐Index to determine the optimal feature set models. Additionally, we evaluate their performance by computing the ROC‐AUCs and Brier scores. The clinical applicability of the three optimal models was evaluated using the DCA. Data Analysis Flowchart See Figure 1.

**TABLE 1:** Basic characteristics of patients with HER2‐positive HR‐negative breast cancer.

Characteristics	Test set (*N* = 1633)	Training set (*N* = 5683)	Validation set (*N* = 803)	Overall (*N* = 8119)
Age				
15–29	17 (1.0%)	5 (0.6%)	79 (1.0%)	79 (1.0%)
30–39	126 (7.7%)	72 (9.0%)	613 (7.6%)	613 (7.6%)
40–49	286 (17.5%)	145 (18.1%)	1508 (18.6%)	1508 (18.6%)
50–59	543 (33.3%)	259 (32.3%)	2606 (32.1%)	2606 (32.1%)
60–69	380 (23.3%)	178 (22.2%)	1934 (23.8%)	1934 (23.8%)
70–79	184 (11.3%)	98 (12.2%)	926 (11.4%)	926 (11.4%)
80+	97 (5.9%)	46 (5.7%)	453 (5.6%)	453 (5.6%)
Race				
Asian or Pacific Islander	1375 (84.2%)	4761 (83.8%)	669 (83.3%)	6805 (83.8%)
White	246 (15.1%)	874 (15.4%)	125 (15.6%)	1245 (15.3%)
American Indian/Alaska Native	12 (0.7%)	48 (0.8%)	9 (1.1%)	69 (0.8%)
Marital				
Divorced	174 (10.7%)	605 (10.6%)	84 (10.5%)	863 (10.6%)
Married	1029 (63.0%)	3597 (63.3%)	505 (62.9%)	5131 (63.2%)
Separated	18 (1.1%)	65 (1.1%)	9 (1.1%)	92 (1.1%)
Single	232 (14.2%)	779 (13.7%)	114 (14.2%)	1125 (13.9%)
Unmarried or Domestic Partner	174 (10.7%)	622 (10.9%)	88 (11.0%)	884 (10.9%)
Widowed	6 (0.4%)	15 (0.3%)	3 (0.4%)	24 (0.3%)
Grade				
Grade I	23 (1.4%)	93 (1.6%)	9 (1.1%)	125 (1.5%)
Grade II	394 (24.1%)	1365 (24.0%)	196 (24.4%)	1955 (24.1%)
Grade III	1198 (73.4%)	4177 (73.5%)	591 (73.6%)	5966 (73.5%)
Grade IV	18 (1.1%)	48 (0.8%)	7 (0.9%)	73 (0.9%)
Hist				
IDC, 8500	1533 (93.9%)	5315 (93.5%)	748 (93.2%)	7596 (93.6%)
ILC, 8520	15 (0.9%)	53 (0.9%)	10 (1.2%)	78 (1.0%)
Mixed IDC and ILC, 8522	29 (1.8%)	84 (1.5%)	10 (1.2%)	123 (1.5%)
Other	56 (3.4%)	231 (4.1%)	35 (4.4%)	322 (4.0%)
Stage				
I	539 (33.0%)	1949 (34.3%)	277 (34.5%)	2765 (34.1%)
II	633 (38.8%)	2233 (39.3%)	321 (40.0%)	3187 (39.3%)
III	341 (20.9%)	1092 (19.2%)	157 (19.6%)	1590 (19.6%)
IV	120 (7.3%)	409 (7.2%)	48 (6.0%)	577 (7.1%)
TCODE				
T0	1 (0.1%)	3 (0.1%)	0 (0%)	4 (0.0%)
T1	681 (41.7%)	2465 (43.4%)	363 (45.2%)	3509 (43.2%)
T2	609 (37.3%)	2166 (38.1%)	288 (35.9%)	3063 (37.7%)
T3	187 (11.5%)	575 (10.1%)	79 (9.8%)	841 (10.4%)
T4	155 (9.5%)	474 (8.3%)	73 (9.1%)	702 (8.6%)
NCODE				
N0	889 (54.4%)	3099 (54.5%)	452 (56.3%)	4440 (54.7%)
N1	505 (30.9%)	1780 (31.3%)	241 (30.0%)	2526 (31.1%)
N2	130 (8.0%)	420 (7.4%)	61 (7.6%)	611 (7.5%)
N3	109 (6.7%)	384 (6.8%)	49 (6.1%)	542 (6.7%)
MCODE				
M0	1513 (92.7%)	5274 (92.8%)	755 (94.0%)	7542 (92.9%)
M1	120 (7.3%)	409 (7.2%)	48 (6.0%)	577 (7.1%)
Surg				
NO	147 (9.0%)	487 (8.6%)	62 (7.7%)	696 (8.6%)
YES	1486 (91.0%)	5196 (91.4%)	741 (92.3%)	7423 (91.4%)
Radiation				
NO	866 (53.0%)	2980 (52.4%)	429 (53.4%)	4275 (52.7%)
YES	767 (47.0%)	2703 (47.6%)	374 (46.6%)	3844 (47.3%)
Chemotherapy				
NO	339 (20.8%)	1191 (21.0%)	162 (20.2%)	1692 (20.8%)
YES	1294 (79.2%)	4492 (79.0%)	641 (79.8%)	6427 (79.2%)
Bone				
NO	1582 (96.9%)	5500 (96.8%)	778 (96.9%)	7860 (96.8%)
YES	51 (3.1%)	183 (3.2%)	25 (3.1%)	259 (3.2%)
Brain				
NO	1624 (99.4%)	5653 (99.5%)	799 (99.5%)	8076 (99.5%)
YES	9 (0.6%)	30 (0.5%)	4 (0.5%)	43 (0.5%)
Liver				
NO	1576 (96.5%)	5503 (96.8%)	783 (97.5%)	7862 (96.8%)
YES	57 (3.5%)	180 (3.2%)	20 (2.5%)	257 (3.2%)
Lung				
NO	1591 (97.4%)	5551 (97.7%)	791 (98.5%)	7933 (97.7%)
YES	42 (2.6%)	132 (2.3%)	12 (1.5%)	186 (2.3%)
Status				
Alive	1298 (79.5%)	4587 (80.7%)	652 (81.2%)	6537 (80.5%)
Dead	335 (20.5%)	1096 (19.3%)	151 (18.8%)	1582 (19.5%)
Time				
Mean (SD)	69.5 (29.4)	69.5 (28.7)	70.6 (29.3)	69.6 (28.9)
Median [Min, Max]	70.0 [3.00, 119]	70.0 [3.00, 119]	72.0 [3.00, 119]	70.0 [3.00, 119]

All calculations and analyses in this study were performed using R 4.1.3 and Python 3.9.13 software. Randomization of the data was performed using sklearn, and means and variances were normalized. A *k*‐test (*k* = 10) was used during model training to ensure accuracy. Finally, we used Python in conjunction with the deep learning framework Theano to run the simulations. All tests were two‐tailed, and the significance criterion was set at *p* < 0.05.

## 3 Results

### 3.1 Baseline Characteristics of the Patients

We reviewed the demographic and clinical characteristics of patients with HER2‐positive HR‐negative breast cancer (Table 1). A total of 8119 female patients were included in this study. The majority of patients were aged 50–59 years (32.1%) and 60–69 years (23.8%). The ethnic distribution of the cases was predominantly Asian or Pacific Islander, with a total of 6805 cases (83.8%). Married patients accounted for 63.2%. Of the total patients, 73.5% had high tumor grade (grade III). The majority of patients had pathology type IDC, 8500. Tumor stages were concentrated in stage I (34.1%) and stage II (39.3%), with a large proportion of patients having T1, T2, N0, N1, and M0 statuses. Of the patients, 91.4% received surgery and 79.2% received chemotherapy. Less than half of the patients (47.3%) received radiotherapy. The most common types of distant metastases were bone metastases (3.2%) and brain metastases (3.2%).

**FIGURE 2:**
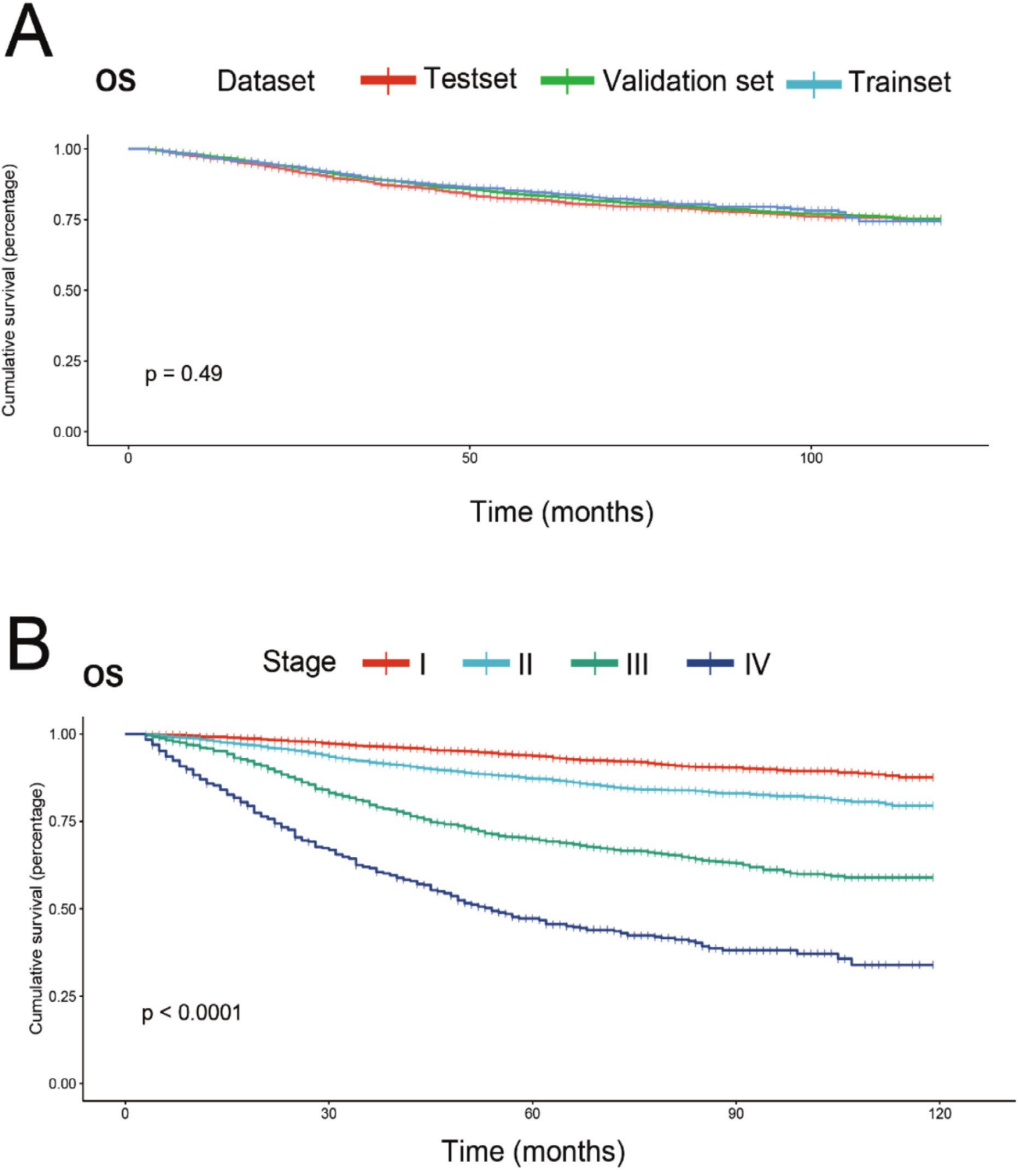
Survival differences between different datasets (A), survival differences between different tumor stages based on overall data (B).

The dataset was divided into three sets: 70% for training, 10% for validation, and 20% for testing. In RSF and Deepsurv, predictive models are generated using the training set, the validation set is used for hyperparameter search, and the remaining 20% is used to evaluate the accuracy and reliability of the model. In CoxPH, the predictive model is generated using the training set, with the validation set, and the test set is used to assess the accuracy and reliability of the model. The basic characteristics of the three data sets are shown in Table 1. There were no differences in overall survival outcomes among the three datasets (Figure 2A). The survival results for tumor staging subgroups in the entire dataset show significant differences (*p* < 0.05), indicating the reliability of our data.

### 3.2 Feature Selection Based on Cox Regression

Using the training set data, we determined a feature set based on Cox regression (Cox 10). Specifically, variables that were statistically significant in the Cox univariate analysis were included in the multivariate analysis. The survival of HER2‐positive HR‐negative breast cancer patients was significantly affected by Stage, Age, M‐code, T‐code, N‐code, Surg, Marital, Chemotherapy, Liver, Brain, Lung, Radiation, Bone, and Hist in univariate analysis (Table 2). In multivariate analysis, stage, age, M‐code, T‐code, N‐code, surgery, marital status, chemotherapy, liver, brain, and lung were identified as prognostic factors for patients with HER2‐positive HR‐negative breast cancer (Table 2).

**TABLE 2:** Univariate analysis of OS in the training cohort.

Variables	OS univariate analysis			OS multivariate analysis		
	95% CI	HR	*p*	95% CI	HR	*p*
Age (vs. 15–29)						
30–39	0.364–1.327	0.695	0.270	0.409–1.523	0.790	0.481
40–49	0.333–1.143	0.618	0.125	0.442–1.551	0.828	0.555
50–59	0.411–1.375	0.752	0.354	0.501–1.747	0.943	0.853
60–69	0.462–1.552	0.848	0.592	0.653–2.26	1.216	0.538
70–79	0.988–3.323	1.812	0.055	1.283–4.477	2.396	0.006**
80+	2.736–9.214	5.021	< 0.001***	2.892–0.395	5.483	< 0.001***
Race (vs. Asian or Pacific Islander)						
White	0.919–2.412	1.489	0.106			
American Indian/Alaska Native	0.699–1.496	1.023	0.908			
Marital (vs. Divorced)						
Married	0.447–0.640	0.536	< 0.001***	0.514–0.738	0.616	< 0.001***
Separated	0.285–1.093	0.558	0.0892.	0.292–0.131	0.574	0.109
Single	0.604–0.947	0.757	0.0151*	0.598–0.945	0.752	0.015*
Unmarried or Domestic Partner	1.446–2.157	1.767	< 0.001***	0.647–1.006	0.807	0.056
Widowed	0.874–4.468	1.976	0.1016	1.419–7.416	3.244	0.005**
Grade (vs. Grade I)						
Grade II	0.676–1.918	1.139	0.625			
Grade III	0.756–2.099	1.259	0.376			
Grade IV	0.577–2.735	1.256	0.566			
Hist (vs. IDC, 8500)						
ILC, 8520	1.076–2.890	1.763	0.024*	0.722–1.964	1.191	0.494
Mixed IDC and ILC, 8522	1.083–2.434	1.623	0.019*	0.773–1.750	1.163	0.470
Other	0.795–1.435	1.068	0.663	0.703–1.278	0.948	0.726
Stage (vs. I)						
II	1.567–2.273	1.887	< 0.001***	1.243–2.291	1.688	< 0.001***
III	3.874–5.589	4.654	< 0.001***	2.343–5.017	3.428	< 0.001***
IV	8.241–12.320	10.076	< 0.001***	2.627–6.589	4.160	< 0.001***
TCODE (vs. T0–1)						
T2	1.868–2.547	2.181	< 0.001***	1.012–1.652	1.298	0.034*
T3	2.925–4.304	3.548	< 0.001***	1.103–1.929	1.459	0.008**
T4	5.461–7.825	6.537	< 0.001***	1.367–2.407	1.813	< 0.001***
NCODE (vs. N0)						
N1	1.683–2.227	1.936	< 0.001***	0.942–1.359	1.132	0.185
N2	2.497–3.670	3.027	< 0.001***	0.912–1.569	1.196	0.196
N3	3.596–5.165	4.309	< 0.001***	1.153–1.929	1.492	0.002**
MCODE (vs. M0)						
M1	4.213–5.653	4.880	< 0.001***	0.439–0.891	0.626	0.009**
Surg (vs. None)						
Yes	0.224–0.280	0.260	< 0.001***	0.438–0.645	0.532	< 0.001***
Rad (vs. None)						
Yes	0.695–0.884	0.784	< 0.001***	0.813–1.067	0.931	0.303
Chemotherapy (vs. None)						
Yes	0.552–0.718	0.63	< 0.001***	0.458–0.630	0.538	< 0.001***
Bone (vs. None)						
Yes	4.031–6.005	4.92	< 0.001***	0.815–1.411	1.072	0.618
Brain (vs. None)						
Yes	4.992–11.85	7.692	< 0.001***	1.612–4.167	2.598	< 0.001***
Liver (vs. None)						
Yes	4.013–5.987	4.902	< 0.001***	1.154–2.003	1.521	0.003**
Lung (vs. None)						
Yes	4.56–185	5.724	< 0.001***	1.053–1.859	1.399	0.020*

*Note:* *Asterisks indicate statistical significance: *p < 0.05, **p < 0.01, ***p < 0.001.

### 3.3 Feature Selection Based on RSF‐Variable Importance

**FIGURE 3:**
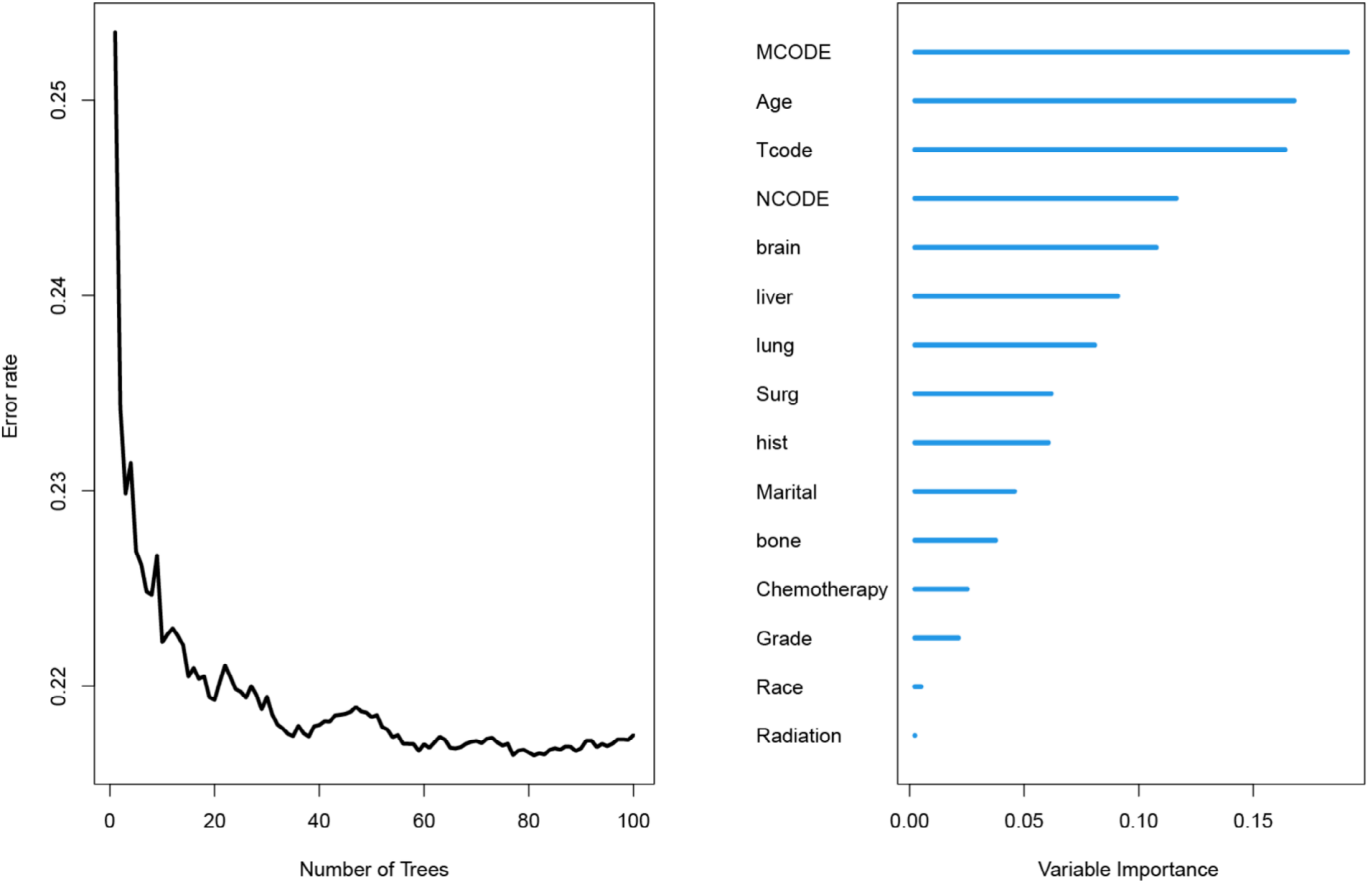
RSF analysis plot and ranking of variable importance. RSF, random survival forest.

We have used RSF‐Variable Importance (RSF‐VIMP) for variable screening. For HER2‐positive/HR‐negative breast cancer patients, the error rate curve in the left panel helped determine the appropriate range of tree numbers for constructing the RSF model to ensure robust predictive performance. In the Figure 3, based on variable importance ranking, we found that the following factors were associated with patient survival outcomes in the following order of importance: MCODE (metastasis status), Age, Tcode (T stage), brain metastasis, liver metastasis, lung metastasis, surgical intervention status, pathological type, marital status, bone metastasis, pathological grade, ethnicity, and radiotherapy status. We selected 12 or 14 variables most associated with prognosis to build the model (RSF12/RSF14).

### 3.4 Feature Selection Based on LASSO Regression

**FIGURE 4:**
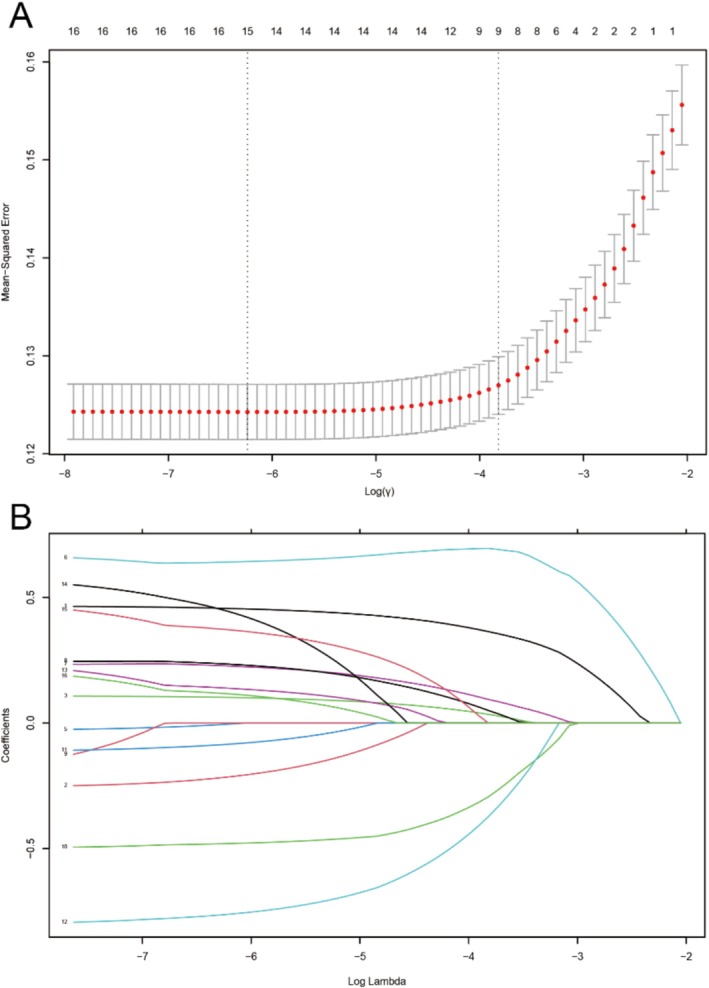
LASSO regression analysis cross‐validation curve (A); LASSO coefficient path diagram (B). LASSO, least absolute shrinkage and selection operator.

In the HER2‐positive/HR‐negative breast cancer cohort, LASSO regression analysis was performed for feature selection (Figure 4). Figure 4A demonstrates the determination of the optimal regularization parameter λ to ensure robust predictive performance of the model. The λ value corresponding to the minimum mean squared error (MSE) was identified as the optimal threshold for feature selection. Figure 4B displays the coefficient trajectories of variables across varying λ values, facilitating the identification of prognostically significant variables. By observing the coefficient shrinkage patterns, we determined which variables were retained at different λ values, ultimately constructing a parsimonious predictive model. Eight features were selected (LASSO‐8) for subsequent analyses: Age, MCODE (metastasis status), T‐code (T stage), NCODE (N stage), Surg (surgical intervention), Marital status, Chemotherapy, and Liver metastasis.

### 3.5 Construct an Optimal Model by Selecting Features

We obtained five sets of feature sets to construct the prognostic models. These sets were generated through LASSO regression (Figure 4), Cox single/multifactor analysis (Table 2), and RSF feature screening (Figure 3) (see Table 3 for details).

**TABLE 3:** Model feature screening process.

Model	Features	Acquisition methods
Lasso8	Age, MCODE, T‐code, NCODE, Surg, Marital, Chemotherapy, liver	LASSO Regression Screening
COX10	Age, MCODE, T‐code, NCODE, Surg, Marital, Chemotherapy, liver, brain, lung	COX single‐factor and multifactor screening
RSF 12	Age, MCODE, T‐code, Surg, hist, NCODE, Marital, Chemotherapy, lung, bone, liver, Race	RSF Feature Importance
RSF14	Age, MCODE, T‐code, Surg, hist, NCODE, Marital, Chemotherapy, lung, bone, liver, Race, brain, Radiation	RSF Feature Importance
ALL 15	Grade, Age, MCODE, Surg, T‐code, hist, lung, NCODE, Marital, bone, Chemotherapy, liver, Race, brain, Radiation	Excluding Stage^a^ features and including all other features results in

^a^ The features of the stage may have multicollinearity potential with the features of T, N, and M.

### 3.6 Deepsurv Model

DeepSurv is a deep feedforward neural network that predicts the impact of patient covariates on patient survival. The structure of the network consists of a large number of simulated neurons divided into three main layers: an input layer, a hidden layer, and an output layer. The neural network architecture consists of a single input layer and a single output layer, with the possibility of having multiple hidden layers. The predictive model was trained using the training data, a hyperparameter search was performed using the validation set and, after several iterations, the optimal learning rate and the minimum loss of values for the algorithm were determined.

### 3.7 The Random Survival Forest (RSF) Model

Two sets of feature sets (RSF12/14) were obtained by ranking the importance of features using RSF modeling. Figure 3 shows the process of variable selection. The optimal structure of the RSF model is determined using a hyperparameter search. It consists of 100 estimators, 10 minimum sample splits, and 10 minimum sample leaves.

### 3.8 Neural Network‐Based Survival Prediction Compared to the Performance of RSF and CoxPH Models

**FIGURE 5:**
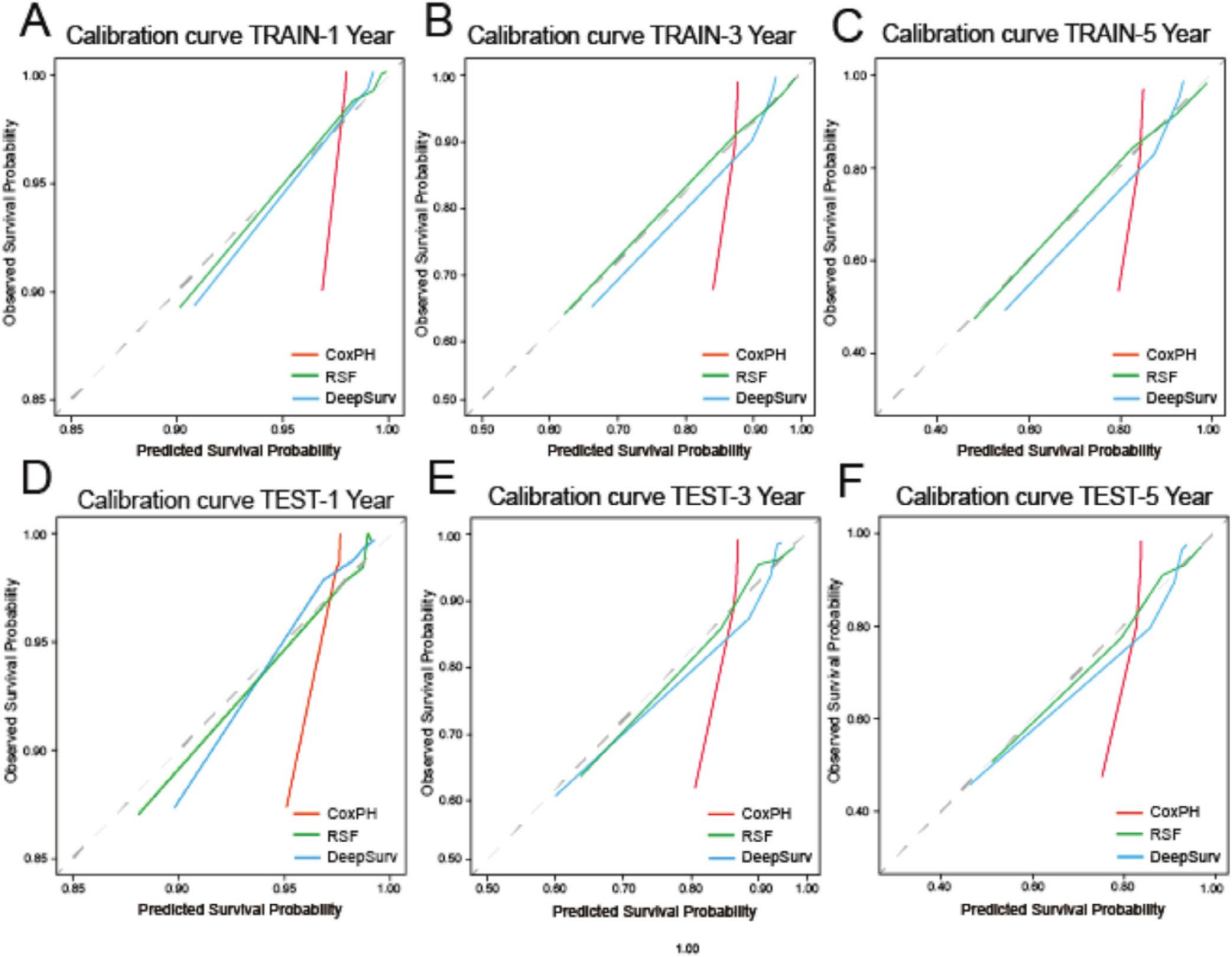
Probability of predicting 1/3/5‐year survival times for the training set (A–C) and validation set (D–F). Gray diagonal lines indicate ideal evaluations, red solid lines indicate the performance of the CoxPH model, green solid lines indicate the DeepSurv model and blue solid lines RSF model predictions at different times.

**FIGURE 6:**
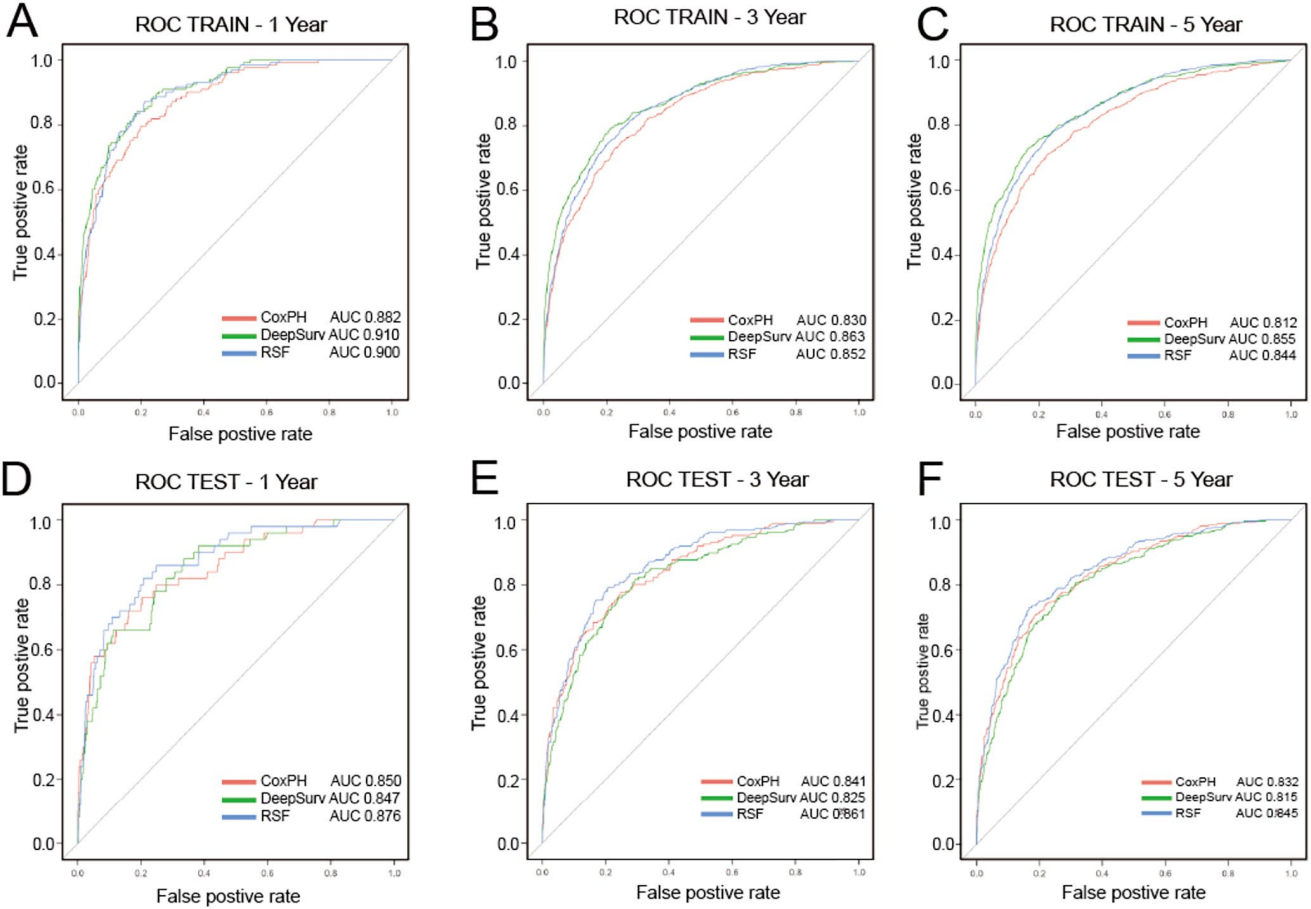
ROC curves for the training set (A–C) and validation set (D–F). Comparing the AUC values of three models for 1‐, 3‐, and 5‐year survival to assess the time‐dependent sensitivity and specificity of the models.

**FIGURE 7:**
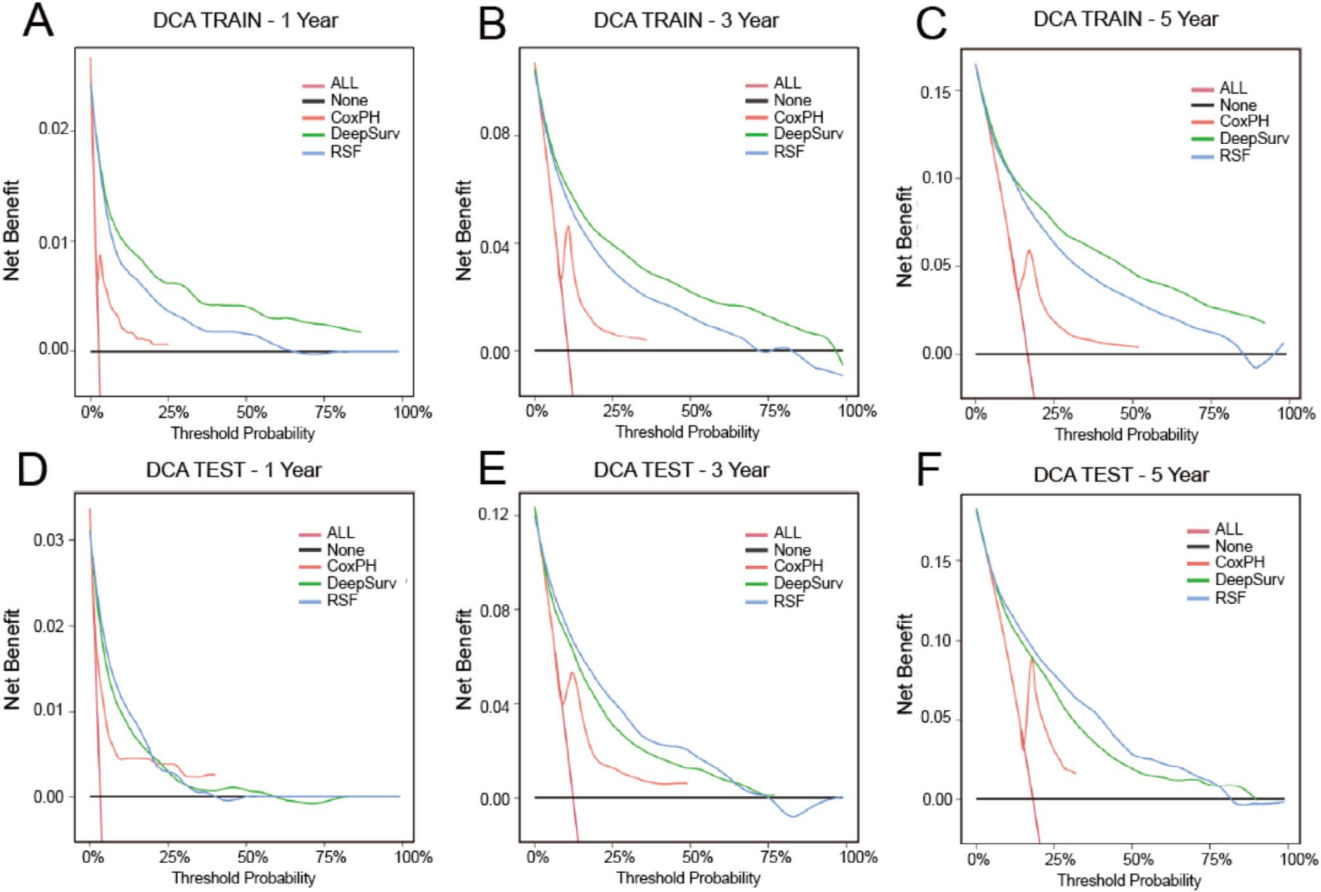
DCA for the training set (A–C) and validation set (D–F). Comparison of DCA for 1‐, 3‐, and 5‐year survival of three models to assess their clinical applicability.

We used LASSO regression, Cox regression, and RSF‐VIMP to select five sets of feature variables for multi‐odel comparative analysis (Table 3). The C‐index of the Deepsurv model constructed based on the training set and various feature sets was all greater than 0.8, which was generally better than the RSF model and the CoxPH model. The Deepsurv model had the highest C‐index of 0.845 when the feature set is RSF14 (obtained by filtering based on RSF features). CoxPH had a higher C‐index when based on the test. However, the Deepsurv model with a feature set of ALL16 (obtained by including all variables except stage) had the highest C‐index of 0.803 (Table 4). The Brier scores of the three models constructed using the optimal elicitation set were less than 0.25, indicating that the models have good accuracy. The ROC‐AUCs of all three models were between 0.7 and 1, indicating that our models have good predictive ability. In the training set, the Deepsurv model had the highest predicted AUC values of 0.91, 0.863, and 0.855 for 1‐, 3‐, and 5‐year overall survival (OS), respectively. The RSF model followed with AUC values of 0.9, 0.852, and 0.844 for the same periods. In the test set, the RSF model predicted the highest AUC values for 1‐, 3‐, and 5‐year OS, with values of 0.876, 0.861, and 0.845, respectively. The CoxPH model followed with AUC values of 0.875, 0.829, and 0.814 (Tables 5 and 6, Figure 6). The calibration plot displays the predictive effect of the three models on overall survival at 1, 3, and 5 years (Figure 5). The DeepSurv model demonstrated the strongest agreement between predictions and actual observations, followed by the RSF model. However, the CoxPH model exhibited poor agreement between predictions and actual observations. In the training set data, the Deepsurv model exhibited the highest net clinical gains at 1, 3, and 5 years, followed by RSF and CoxPH. In the test set data, the RSF model's DCA showed the highest net clinical benefit at 1, 3, and 5 years, followed by Deepsurv and CoxPH (Figure 7).

**TABLE 4:** Evaluating the C‐index of various models with different numbers of variables using training and validation data sets.

	Training			Test		
	RSF	CoxPH	Deepsurv	RSF	CoxPH	Deepsurv
Lasso8	0.789	0.791	0.817	0.785	0.796	0.789
COX10	0.787	0.791	0.818	0.786	0.796	0.794
RSF 12	0.789	0.792	0.840	0.786	0.801	0.767
RSF14	0.791	0.792	0.845	0.787	0.801	0.769
ALL 16	0.790	0.792	0.829	0.786	0.803	0.787

**TABLE 5:** Evaluation of AUC and Brier score values for various models (Training set).

Training set model	AUC			Brier score		
	1 years	3 years	5 years	1 year	3 years	5 years
RSF	0.9	0.852	0.844	0.009	0.037	0.060
CoxPH	0.875	0.829	0.814	0.009	0.061	0.097
Deepsurv	0.910	0.863	0.855	0.029	0.087	0.116

**TABLE 6:** Evaluation of AUC and Brier score values for various models (Test set).

Test set model	AUC			Brier score		
	1 years	3 years	5 years	1 year	3 years	5 years
RSF	0.876	0.861	0.845	0.011	0.033	0.037
COXPH	0.850	0.841	0.832	0.011	0.041	0.067
Deepsurv	0.847	0.825	0.815	0.019	0.067	0.091

## 4 Discussion

In this study, we employed three methodologies—the CoxPH model, RSF model, and DeepSurv model—to predict long‐term survival outcomes for a specific breast cancer subpopulation using data from the SEER database. Model performance was systematically compared by integrating C‐index with three distinct feature selection approaches: LASSO regression, Cox regression, and RSF‐VIMP. Additionally, we identified the most impactful feature selection schemes across the models. Our findings aim to provide insights into optimal predictive modeling strategies for breast cancer survival analysis in clinical research.

The primary objective of this study was to evaluate the performance of the aforementioned methods. The most notable finding from the analysis revealed that the RSF‐based method outperformed the DeepSurv model and Cox model in predicting event risk for breast cancer patients. However, the selection of feature sets significantly influenced model performance. Among these, the RSF14 feature set (derived from RSF‐VIMP) significantly improved the performance of all models, highlighting the critical role of feature selection in nonlinear models.

These findings align with previous studies on breast cancer in the literature. Buyrukoğlu et al. [12] constructed survival analysis models using the GBSG2 and METABRIC databases and compared the CoxPH model, RSF model, and Conditional Inference Forest (Cforest) model. Their results demonstrated that RSF and Cforest achieved superior C‐indices of 0.74 and 0.74, respectively, outperforming the traditional Cox model in predicting breast cancer survival probabilities. Similarly, Wang et al. [13] extracted data from the SEER database for metastatic laryngeal and hypopharyngeal cancer patients and developed an RSF model to predict survival outcomes. The model exhibited robust performance, with time‐dependent AUC values exceeding 0.8 in both training and testing sets, underscoring the efficacy of RSF in survival prediction. In another study, Li et al. [14] established an RSF model based on real‐world single‐center data to predict prognosis for HER2‐low breast cancer patients, achieving AUC values of 0.726, 0.712, and 0.685 in the testing set, thereby providing clinicians with a valuable prognostic tool. Additionally, Ren et al. [15] utilized the SEER database to compare long‐term outcomes between breast‐conserving surgery with radiotherapy and mastectomy in early‐stage breast cancer patients following neoadjuvant systemic therapy. Their RSF model achieved C‐indices of 0.847 and 0.795 in the training and validation sets, significantly surpassing other models. However, most existing studies rely solely on the C‐index as the primary performance metric, whereas our study incorporates a comprehensive evaluation framework combining the C‐index, ROC curves, Brier score, and DAC curves. In addition to this, our findings (Table 3) align with prior studies, confirming that long‐term survival in breast cancer is primarily associated with age, tumor size, lymph node metastasis status, surgical approach, and chemotherapy [16, 17]. These results demonstrate that the RSF model, similar to the CoxPH model (which identifies predictors based on *p* values), can effectively identify key factors for early recurrence risk through VIMP. In our study, DeepSurv exhibited optimal performance on the training set (C‐index > 0.8, AUC = 0.91) but underperformed on the test set (C‐index = 0.769 vs. RSF's 0.787; AUC = 0.847 vs. RSF's 0.876). Our findings are consistent with those of Paris et al. [18] indicating that machine learning models may experience overfitting when: (1) they exhibit excellent performance on the training set but a significant decline on the test set; (2) test‐set performance metrics (e.g., AUC values) are substantially lower than those obtained from the overall dataset; or (3) model complexity mismatches the data scale. The CoxPH model demonstrated acceptable performance in terms of C‐index (training set: C‐index = 0.796, test set: C‐index = 0.803) but performed the poorest in AUC and calibration capability. In contrast, the RSF model achieved optimal performance on the test set (training set: C‐index = 0.791, test set: C‐index = 0.787), along with the best AUC, calibration capability, and highest net benefit in DCA. Furthermore, the RSF model demonstrates unique strengths in variable importance evaluation. By calculating VIMP, the RSF model identifies key predictors with the greatest impact on outcomes. The RSF algorithm was first proposed in 2008 and has since been used as a baseline model in many model comparisons. RSF has advantages over traditional Cox regression analysis when dealing with high‐dimensional data [19]. Although the deepsurv model consistently performs well, its “black box” feature remains an obstacle [11]. The RSF algorithm strikes a balance between model fitting and interpretation [9]. As demonstrated in this study, the RSF model exhibits the best fit and overall performance across different datasets. The ongoing evolution of artificial intelligence (AI) in oncology is poised to drive substantial advancements in the predictive capabilities of machine learning‐based prognostic models [20].

It is worth noting that this study has some limitations. First, the data in this study were extracted from the same database and lacked external data validation, which may reduce the generalizability of the model. Second, due to the limitations of the SEER database, some variables that may affect survival, such as type of chemotherapy, genetic factors, etc., were not available. Adding additional potential variables may improve the performance of the RSF model. At the same time, the DeepSurv model has its inherent limitations in the construction process. The presence of hidden layers in the black‐box model means that we cannot accurately understand the computations performed during the model construction process or the limitations associated with them. The use of all‐cause mortality as an end‐point is flawed. If such a model is to be used in decision making then therapies are targeted as the cancer‐specific mortality, so future models must be able to specifically predict cancer‐specific mortality. Our future research should attempt to address the above issues.

## 5 Conclusions

In conclusion, compared to conventional prognostic models, the RSF model serves as a reliable tool for predicting long‐term survival in breast cancer patients, demonstrating consistent performance across diverse datasets. Furthermore, the feature set selected via RSF‐ VIMP (compared with LASSO regression and Cox regression) significantly enhances the performance of prognostic models. Our findings may offer practical guidance for future development of long‐term prognostic models tailored to breast cancer subtypes.

## Author Contributions

**Wenqi Cai:** conceptualization (lead), data curation (lead), formal analysis (lead), funding acquisition (equal), investigation (equal), methodology (equal), project administration (equal). **Yan Qi:** conceptualization (equal), data curation (equal), formal analysis (equal), investigation (lead), methodology (lead). **Linhui Zheng:** investigation (lead), resources (lead), software (equal), supervision (equal), writing – original draft (equal). **Huachao Wu:** supervision (equal), validation (equal). **Chunqian Yang:** visualization (equal), writing – review and editing (equal). **Runze Zhang:** funding acquisition (equal), investigation (equal). **Chaoyan Wu:** conceptualization (equal), data curation (equal), supervision (lead), validation (lead), writing – original draft (equal), writing – review and editing (equal). **Haijun Yu:** methodology (equal), project administration (equal), resources (equal), software (equal), supervision (lead), validation (lead), visualization (lead), writing – original draft (lead), writing – review and editing (lead).

## Conflicts of Interest

The authors declare no conflicts of interest.

## Data Availability

The data used in this analysis may be requested from the National Cancer Institute, Surveillance, Epidemiology, and End Results Program at https://seer.cancer.gov/data/access.html.
